# Differential Expression of a Panel of Ten CNTN1-Associated Genes during Prostate Cancer Progression and the Predictive Properties of the Panel towards Prostate Cancer Relapse

**DOI:** 10.3390/genes12020257

**Published:** 2021-02-10

**Authors:** Yan Gu, Mathilda Jing Chow, Anil Kapoor, Xiaozeng Lin, Wenjuan Mei, Damu Tang

**Affiliations:** 1 Department of Surgery, McMaster University, Hamilton, ON L8S 4K1, Canada; guy3@mcmaster.ca (Y.G.); mathildachow1994@gmail.com (M.J.C.); akapoor@mcmaster.ca (A.K.); linx36@mcmaster.ca (X.L.); wenjuanmei1986@gmail.com (W.M.); 2 Urological Cancer Center for Research and Innovation (UCCRI), St Joseph’s Hospital, Hamilton, ON L8N 4A6, Canada; 3 The Research Institute of St Joe’s Hamilton, St Joseph’s Hospital, Hamilton, ON L8N 4A6, Canada

**Keywords:** prostate cancer, prostate cancer recurrence, contactin 1, biomarkers

## Abstract

Contactin 1 (CNTN1) is a new oncogenic protein of prostate cancer (PC); its impact on PC remains incompletely understood. We observed CNTN1 upregulation in LNCaP cell-derived castration-resistant PCs (CRPC) and CNTN1-mediated enhancement of LNCaP cell proliferation. CNTN1 overexpression in LNCaP cells resulted in enrichment of the CREIGHTON_ENDOCRINE_THERAPY_RESISTANCE_3 gene set that facilitates endocrine resistance in breast cancer. The leading-edge (LE) genes (*n* = 10) of this enrichment consist of four genes with limited knowledge on PC and six genes novel to PC. These LE genes display differential expression during PC initiation, metastatic progression, and CRPC development, and they predict PC relapse following curative therapies at hazard ratio (HR) 2.72, 95% confidence interval (CI) 1.96–3.77, and *p* = 1.77 × 10^−9^ in The Cancer Genome Atlas (TCGA) PanCancer cohort (*n* = 492) and HR 2.72, 95% CI 1.84–4.01, and *p* = 4.99 × 10^−7^ in Memorial Sloan Kettering Cancer Center (MSKCC) cohort (*n* = 140). The LE gene panel classifies high-, moderate-, and low-risk of PC relapse in both cohorts. Additionally, the gene panel robustly predicts poor overall survival in clear cell renal cell carcinoma (ccRCC, *p* = 1.13 × 10^−11^), consistent with ccRCC and PC both being urogenital cancers. Collectively, we report multiple CNTN1-related genes relevant to PC and their biomarker values in predicting PC relapse.

## 1. Introduction

Prostate cancer (PC) ranks one of the top commonly diagnosed cancer and the second leading cause of cancer death in men in the developed world [1]. Organ-confined PCs can be managed by active surveillance, prostatectomy, and radiation. Treatment decisions are based on several factors such as disease severity, and patient age and preference. The disease severity is commonly graded with Gleason score (GS) and GS-based grading system, World Health Organization (WHO) PC grading system (WHO grade group I-V) or ISUP (the International Society of Urological Pathology) grade [2,3,4]. At the molecular level, PCs can be classified into three integrative clusters (iClusters), iCluster 1, iCluster 2, and iCluster 3 [5]; this classification is based on alterations occurred in genome, methylation, and gene expression [6]. While iCluster 1 PCs are enriched with *TMPRSS2-ETV1* and *TMPRSS2-ETV4* fusions, iCluster 2 PCs are featured with *TMPRSS2-ERG* fusion [5]. Fusion of the androgen receptor (AR) target gene *TMPRSS2* to the ETS (E26 transcription-specific) gene family (*ERG*, *ETV1*, and *ETV4*), particularly the *TMPRSS2-ERG*, commonly occurs in PC and plays important role in PC initiation and progression [7,8]. While PC severity can facilitate therapy decision making, current management of PC patients needs significant improvement; as approximately 30% of patients undertaking the curative surgery treatment will have PC relapse or biochemical recurrence (BCR), evident by increases in serum prostate-specific antigen (PSA) [9]. The relapsed PCs are at significantly higher risk of metastasis [10]. Metastatic PCs (mPCs) are commonly managed with androgen deprivation therapy (ADT); resistance nonetheless commonly develops in the form of castration-resistant PC (CRPC) [11,12]. Although multiple therapeutic options are available for CRPCs, including taxane-based chemotherapy [13], AR-targeting therapy involving either abiraterone or enzalutamide [12,14,15,16], and immunotherapy [17,18], effective control of CRPC remains the major challenge [12,19,20]. This reflects the complex mechanisms underlying PC progression.

Contactin 1 (CNTN1) is an Ig-like neural cell adhesion protein [21]; it plays an important role in the central nervous system through promoting axon elongation in the cerebellum, formation of the septate-like junctions between axons and myelinating glial cells, and generation of the neuromuscular junction [22,23,24]. Recent evidence clearly reveals CNTN1-derived oncogenic actions [25]. CNTN1 facilitates lung cancer metastasis [26] and associates with poor prognosis in patients with lung, esophageal and oral squamous cell carcinomas [26,27,28], and hepatocellular carcinoma [29]. CNTN1 upregulation and its association with worse clinical features have been reported in breast cancer [30], astrocytic glioma [31], thyroid cancer [32], and stomach cancer [33]. In prostate cancer, CNTN1 upregulation occurs in cancer stem cells and primary cancer [34]. Functionally, CNTN1 promotes PC metastasis [34]. Clinically, CNTN1 is associated with PC relapse [34,35]. Nonetheless, CNTN1 is a relatively new oncogenic protein, particularly in PC; its contributions to PC progression remain largely uncharacterized.

We report here CNTN1 upregulation in LNCaP cell-derived xenografts produced in castrated mice compared to the xenografts generated in intact mice. Ectopic expression of CNTN1 in LNCaP cells enhances cell proliferation. RNA-sequencing analysis of LNCaP empty vector (EV) cells and LNCaP CNTN1 cells identified an enrichment of the CREIGHTON_ENDOCRINE_THERAPY_RESISTANCE_3 gene set in LNCaP CNTN1 cells. The 10 leading-edge genes of this enrichment are novel to PC; they display differential expression in PC compared to normal prostate tissue, PCs with *TMPRSS2-ERG* fusion compared to those without the fusion, and mPCs compared to primary PC. Furthermore, this 10-gene panel robustly predicts PC relapse in two independent cohorts, the TCGA PanCancer (*n* = 492) and Memorial Sloan Kettering Cancer Center (MSKCC, *n* = 140), as well as poor overall survival (OS) in another urogenital cancer, clear cell renal cell carcinoma (ccRCC). Collectively, we observed a novel aspect of CNTN1-associated genes; this research is promising not only in our understanding of PC but also in improving the clinical management of PC patients.

## 2. Materials and Methods

### 2.1. Cell Culture

LNCaP cells were purchased from American Type Culture Collection (ATCC) and cultured in RPMI1640 with supplementation of 10% FBS (Sigma Aldrich, Oakville, ON, Canada) and 1% Penicillin-Streptomycin (Thermo Fisher Scientific, Burlington, ON, Canada). The cell line was authenticated (Cell Line Authentication Service, ATCC), and routinely checked for Mycoplasma contamination (a PCR kit from Abm, Cat#: G238). LNCaP empty vector (EV) and CNTN1 stable lines were constructed using retrovirus following our established conditions [34,36,37,38]. Briefly, a gag-pol, an envelope (VSV-G; Stratagene), and a designed retroviral vector (EV or CNTN1) were transiently co-transfected into HEK293T cells at the ratio 1:1:1. At 48 h post transfection, the virus-containing medium was filtered (0.45 µm filter) and centrifuged (50,000× *g*) for 90 min to concentrate the viral vectors. The retrovirus pellets were resuspended into RPMI1640 medium and LNCaP cells were subsequently infected and selected for stable integration with puromycin (1 µg/mL; Sigma, Oakville, ON, Canada).

### 2.2. Western Blot Analysis

Cells lysates were prepared in a lysate buffer consisting of 20 mM Tris (pH 7.4), 150 mM NaCl, 1 mM ethylenediaminetetraacetic acid (EDTA), 1 mM EGTA, 1% Triton X-100, 25 mM sodium pyrophosphate, 1 mM NaF, 1 mM β-glycerophosphate, 0.1 mM sodium orthovanadate, 1 mM phenylmethylsulfonyl fluoride (PMSF), 2 µg/mL leupeptin, and 10 µg/mL aprotinin. A total of 50 µg of whole cell lysate was separated on SDS-PAGE gel, and transferred onto Hybond ECL nitrocellulose membranes (Amersham, Little Chalfont, UK), followed by blocking with 5% skim milk at room temperature for one hour. Primary antibodies were incubated overnight at 4 °C with agitation, and secondary antibodies incubated for one hour at room temperature. Signals were then developed (ECL Western blotting Kit, Amersham, Little Chalfont, UK). Primary antibodies used were anti-CNTN1 (1:200; R&D Systems, Minneapolis, MI, USA) and anti-Actin 1:1000 (Santa Cruz Biotechnology, Dallas, TE, USA).

### 2.3. RNA Sequencing Analysis

RNA sequencing analysis was carried out following our established conditions [39]. RNA was extracted from LNCaP cells (stably expressing EV or CNTN1) using a miRNeasy Mini Kit (Qiagen, Toronto, ON, Canada; No. 217004) according to the manufacturer’s instructions; libraries were prepared with TruSeq Ribo Profile Mammalian Kit (Illumina, San Diego, CA, USA; RPHMR12126) following manufacturer’s instructions, and sequenced by BGI using the HiSeq 4000 system. RNA sequences were quantified by BGI (http://www.genomics.cn/en/navigation/show_navigation?nid=2657 accessed on 19 December 2020) and expressed as fragments per kilobase of exon per million fragments mapped (FPKM) according to the formula: FPKM = 10^6^C/(NL/10^3^) with C being the number of fragments aligned to a specific gene, N representing the total number of fragments aligned, and L for the combined exon length of a gene. Differentially expressed genes (DEGs) were produced by BGI using the Poisson Distribution Method.

### 2.4. Analysis of CNTN1-Associated Genes

Fast Gene set enrichment analysis (FGSEA) on CNTN1-associated DEGs was performed using Galaxy (https://usegalaxy.org/ accessed on 19 December 2020). Gene expression in PC and normal prostate tissues was carried out using GEPIA2 database [40]. Analyses of gene expression in mPCs and primary PCs were determined with using R2: Genomics Analysis and Visualization Platform (http://r2.amc.nl http://r2platform.com accessed on 19 December 2020). A curated GEO dataset (GDS2546: GEO DataSet Browser (nih.gov accessed on 19 December 2020)) was also used to analyze leading-edge (LE) gene expression following PC progression.

### 2.5. Generation of CRPC in Animal Models

Xenografts were generated as previously described. In brief, LNCaP cells (5 × 10^6^) in 0.1 mL media were mixed with Matrigel mixture (BD) at 1:1 ratio and implanted subcutaneously (s.c.) into the flank of NOD/SCID mice (6-week-old males; The Jackson Laboratory). Tumor growth were monitored through palpation and measured twice weekly using calipers. Tumor volume was calculated as V = L × W^2^ × 0.52 [41]. Tumor progression was monitored by serum PSA levels (PSA kit, Abcam, Toronto, ON, Canada). Surgical castration was performed when tumor reached 100–200 mm^3^ in size. Serum PSA was determined before and after castration. Resurge in serum PSA indicates CRPC growth.

### 2.6. Patient Populations

cBioPortal [42,43] (https://www.cbioportal.org/ accessed on 19 December 2020) contains the most well-organized and comprehensive cancer genetic data for different cancer types. The TCGA (The Cancer Genome Atlas) PanCancer PC dataset contains 492 PCs and MSKCC contains 140 PCs with follow-up data, respectively. The data sets were used to analyze the LE multigene panel biomarker values in predicting PC recurrence. The TCGA PanCancer ccRCC (clear cell renal cell carcinoma) dataset consists of 512 patients with primary ccRCC. Both PC and ccRCC TCGA PanCancer datasets have been well-demonstrated for its suitability in survival (relapse for PC and overall survival for ccRCC) biomarker studies [44].

### 2.7. Assignment of Signature Scores to Patients/Tumors and Cutoff Point Estimation

All component genes in the LE multigene panel were examined for an association with PC progression (relapse) using multivariate Cox proportional hazards (PH) regression with the R survival package. The panel scores for individual patients were given using Sum (coef_1_ × gene_1exp_ + coef_2 ×_ gene_2exp_ + … …+ coef_n_ × gene_nexp_, *n* = 10), where coef_1_ … coef_n_ are the coefs of individual genes and gene_1exp_ … … gene_nexp_ are the expression of individual genes. Cutoff point to stratify tumors with high and risk of PC relapse was estimated using Maximally Selected Rank Statistics (the Maxstat package) in R.

### 2.8. Statistical Analysis

Kaplan–Meier surviving curves, log-rank test, and Cox proportional hazards (Cox PH) regression analyses were carried out using the R survival package. The PH assumption was tested. Univariate and multivariate Cox regression analyses were run using the R survival package. Time-dependent receiver operating characteristic (tROC) analysis was performed using the R timeROC package. A two-tailed Student’s t test, one-way or two-way analysis of variance (ANOVA) and Tukey’s multiple comparisons post-hoc test were also performed. A value of *p* < 0.05 is considered statistically significant.

## 3. Results

### 3.1. CNTN1-Mediated Promotion of LNCaP Cell Proliferation and Effects on Gene Expression

**Figure 1:**
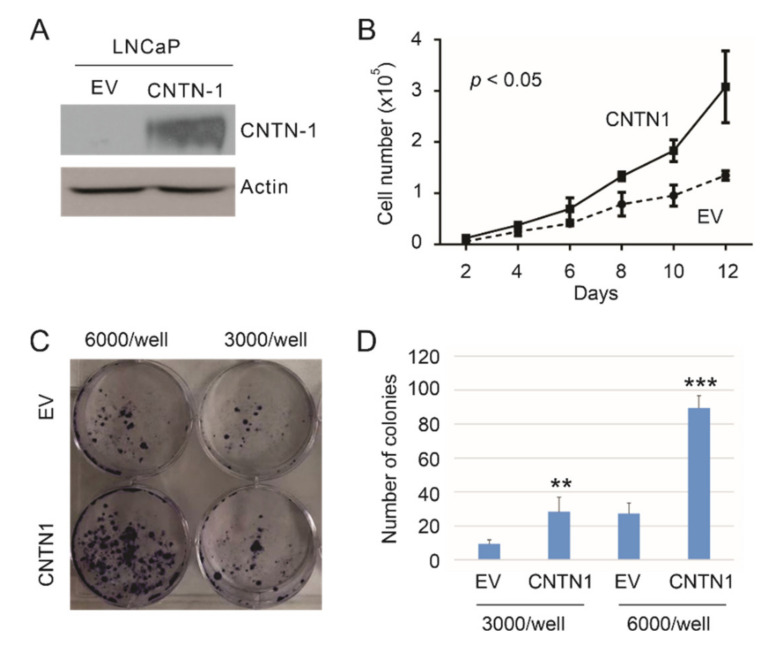
CNTN1 enhances LNCaP cell proliferation. (**A**) Western blot analysis for CNTN1 and Actin expression in the indicated stable lines. (**B**) LNCaP empty vector (EV) and LNCaP CNTN1 cells were seeded at 10^4^/well in 6-well plate. Cell numbers were counted every two days. Experiments were repeated three times. Mean ± SD (standard deviation) was graphed; 2-way ANOVA was used to analyze the two growth curves. (**C**,**D**) LNCaP EV and LNCaP CNTN1 cells were seeded in 60 mm plates at the indicated densities for colony formation. Experiments were repeated three times. Typical images from a single repeat are presented (**C**); quantifications were graphed using Mean ± SD. ** *p* < 0.01, *** *p* < 0.001 in comparison to the respective EV control by 2-tailed Student t-test (**D**).

We have observed that CNTN1 promoted DU145 cell-derived xenograft formation and lung metastasis along with facilitating AKT activation [34]; CNTN1 also enhanced the invasive ability of C4-2 cell, a derivative of LNCaP cell, in vitro [34]. To further examine CNTN1′s oncogenic actions in PC, we stably expressed an empty vector (EV) or CNTN1 in LNCaP cells (Figure 1A). In comparison to LNCaP EV cells, LNCaP CNTN1 cells display increases in cell proliferation (Figure 1B) and colony formation (Figure 1C,D), supporting CNTN1 enhancing LNCaP cell proliferation.

**Figure 2:**
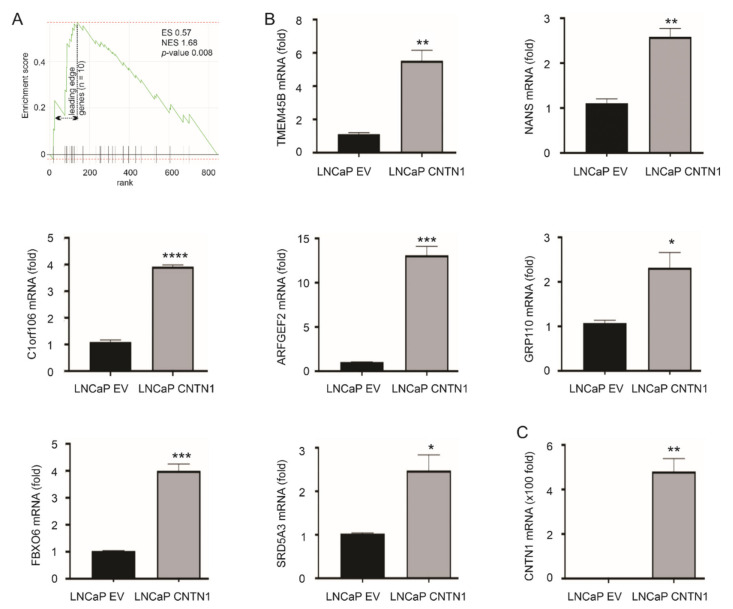
Enrichment of CREIGHTON_ENDOCRINE_THERAPY_RESISTANCE_3 gene set in LNCaP CNTN1 cells. (**A**) Gene set enrichment analysis (GSEA) analysis reveals the enrichment of CREIGHTON_ENDOCRINE_THERAPY_RESISTANCE_3 gene set in the differentially expressed genes (DEGs) relative to CNTN1 overexpression (LNCaP CNTN1 vs. LNCaP EV). The leading-edge genes (*n* = 10) in this enrichment are indicated (see Table 1 for individual leading-edge genes). (**B**) Confirmation of the upregulations of 7 of 10 leading-edge genes in LNCaP CNTN1 cells. (**C**) Confirmation of CNTN1 overexpression in LNCaP CNTN1 cells. * *p* < 0.05, ** *p* < 0.01, *** *p* < 0.001, **** *p* < 0.0001.

To further examine the major pathways affected by CNTN1, we performed RNA-sequencing analysis on LNCaP EV and LNCaP CNTN1 cells. A set of differentially expressed genes (DEGs) were identified between LNCaP EV and LNCaP CNTN1 cells (see Section 2.3). To further analyze these DEGs, we performed gene set enrichment analysis using FGSEA (see Section 2.4) and the “C2: curated gene sets” (Broad Institute, Cambridge, MA, USA) [45], the largest gene set collection (*n* = 6226) within the MSsigDB collection (http://www.gsea-msigdb.org/gsea/msigdb/collections.jsp# accessed on 19 December 2020). The analysis identified the CREIGHTON_ENDOCRINE_THERAPY_RESISTANCE_3 gene set being enriched (Figure 2A). This gene set is associated with endocrine therapy resistance in breast cancer [46]. This resistance, which arose from therapies targeting estrogen receptor signaling, likely shares similarities with resistance to androgen-deprivation therapy (ADT) because of the commonalities between androgen and estrogen with respect to their synthesis and signalling. We thus have focused on investigating the relevance of this enrichment in PC. The enrichment has a leading-edge gene set of 10 genes (Figure 2A, Table 1); the leading-edge subset genes were defined by enrichment score with the maximal distance from the baseline (zero) (Figure 2A) [45]. Except *LY75*, *RNF144A*, and *PTPN3*, we were able to confirm the upregulation of other 7 LE genes: *TMEM45B*, *NANS*, *C1orf106*, *ARFGEF2*, *GPR110*, *FBXO6*, and *SRD5A3* in LNCaP CNTN1 cells compared to LNCaP EV cells (Figure 2B). The overexpression status of CNTN1 in LNCaP CNTN1 cells was confirmed (Figure 2C).

### 3.2. Differentional Expression of the 10 Leading-Edge Genes Following PC Progression

Leading-edge genes associated with the enrichment are likely important with respect to CNTN1-affected PC pathogenesis. The 10 leading-edge (LE) genes include *TMEM45B* [47], *NANS* [48], *GPR110* [49], and *SRD5A3* [50,51] which are related to PC and AR signaling (Table 1). SRD5A3 sustains androgen biosynthesis and contributes to CRPC [50,51]. The inclusion of these four genes in LE genes supports the relevance of these LE genes in PC tumorigenesis and progression. Nonetheless, these LE genes are novel to PC, evident by 3 of 10 LE genes being unknown in PC (Table 1). With respect to LY75 and RNF144A, althogh both proteins were detected in PC tissues based on information presented in “Human Protein Atlas” (https://www.proteinatlas.org/ accessed on 19 December 2020), there are no articles listed in PubMed under the search term “LY75 and prostate cancer” and “RNF144A and prostate cancer” as of 23 January 2021; their involvement in PC thus remains unclear.

**Table 1:** The leading-edge (LE) genes and their function in prostate cancer (PC) and tumorigenesis.

Gene	Gene Details	Log2 ^1^	*p*-Value	Function in PC	Refs
*TMEM45B*	transmembrane protein 45B	12.2	0.0015 **	a biomarker of PC progression and metastasis	[47]
*NANS*	N-acetylneuraminic acid synthase	11.9	0.0428 *	an androgen-responsive gene	[48]
*C1orf106*	chromosome 1 open reading frame 106	10.2	0.0428 *	unknown	NA
*LY75*	lymphocyte antigen 75	9.8	0.0164 *	not clear	NA
*ARFGEF2*	ADP-ribosylation factor guanine nucleotide-exchange factor 2	9.5	0.0101 *	protein detected in PC bone metastasis	[52]
*RNF144A*	ring finger protein 144A	3.2	0.0028 **	not clear	NA
*GPR110*	G protein-coupled receptor 110	3.0	0.0152 *	an oncogene in PC	[49]
*FBXO6*	F-box protein 6	2.9	1.06 × 10^−5^ ***	unknown	NA
*PTPN3*	protein tyrosine phosphatase, non-receptor type 3	2.6	0.0065 **	unknown	NA
*SRD5A3*	steroid 5 α-reductase 3	2.3	0.0027 **	sustaining androgen biosynthesis in CRPC	[50,51]

^1^ Log2 Ratio: CNTN1/EV; * *p* < 0.05, ** *p* < 0.01, *** *p* < 0.001. CRPC: castration-resistant prostate cancer.

**Figure 3:**
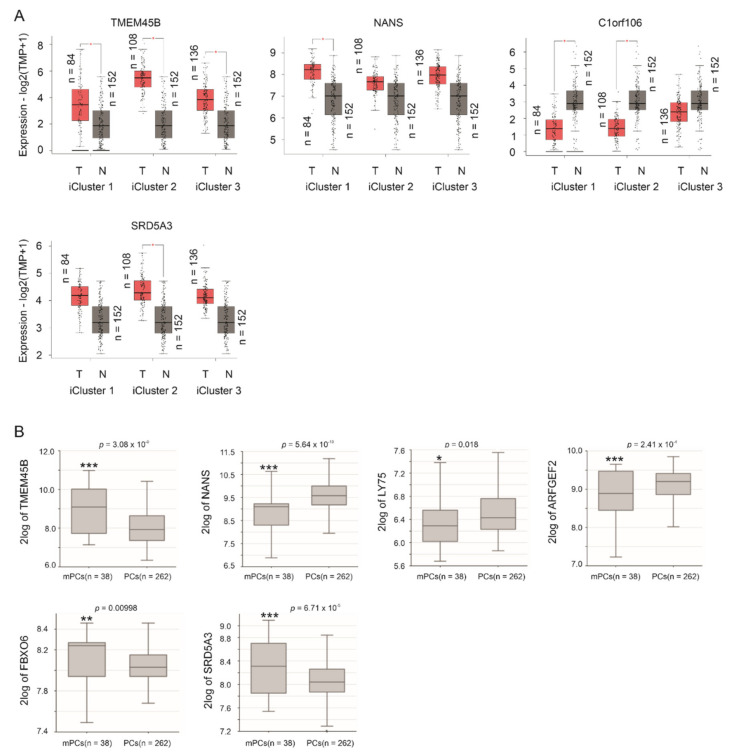
Differential expression of LE genes in PCs and mPCs compared to prostate tissues and primary PCs, respectively. (**A**) The analyses were performed using the GEPIA2 database. T: tumor (PC), N: normal prostate tissues, and TPM: transcripts per million. Statistical analyses were performed by GEPIA2, * *p* < 0.05. (**B**) Analyses were performed using the Sawyers dataset in R2: Genomics Analysis and Visualization Platform. Expressions were presented as log2-transformed data. Statistical analyses were performed by the R2 Platform using one-way ANOVA. * *p* < 0.05; ** *p* < 0.01; *** *p* < 0.001.

By using the GEPIA2 database [40], we demonstrated TMEM45B upregulation in all three iCluster PCs compared to prostate tissues, C1orf106 downregulation in iCluster 1 and iCluster 2 PCs compared to prostate tissues, NANS increase in iCluster 1 PCs, and SRD5A3 upregulation in iCluster 2 PCs compared to prostate tissues (Figure 3A). PCs within iCluster 1 are featured with ETV1 and ETV4 fusion, SHOP mutations, FOXA1 mutations, and CHD1 deletion, but lack ERG fusion [5]. iCluster 2 PCs are enriched with ERG fusion and PTEN deletion [5]. iCluster 3 PCs contain ERG fusion [5]. TP53 hetero-deficiency and RB1 deletion occur more frequently in iCluster 1 and iCluster 2 PCs [5]. Advanced PCs (GS ≥ 8) are much more common in iCluster 1 and iCluster 2 compared to iCluster 3 [5], suggesting that PCs in iClusters 1 and 2 are more aggressive than those in iCluster 3. This concept indicates an association of the 10 LE genes with PC progression.

**Figure 4:**
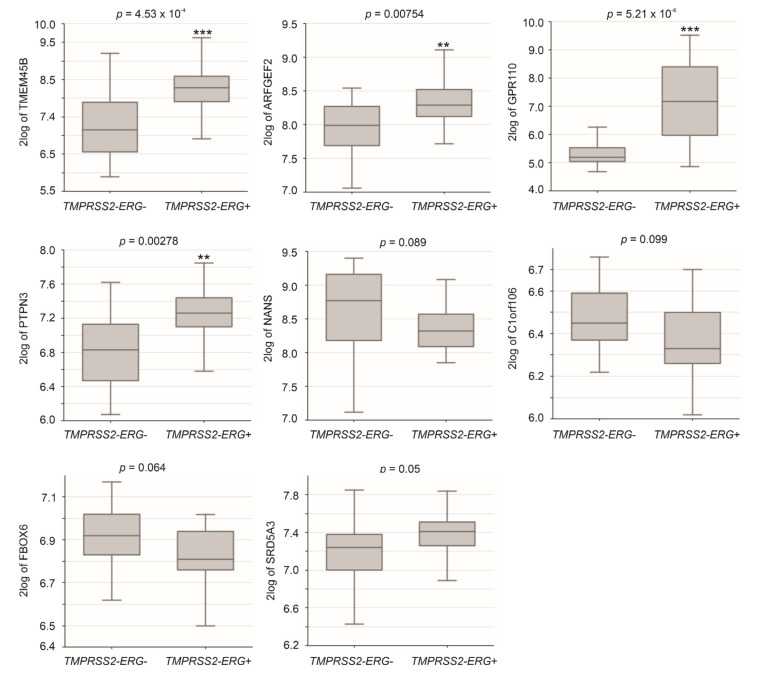
Differential expression of LE genes in PCs with *TMPRSS2-ERG* fusion. Analyses were performed using the Sueltman dataset in R2: Genomics Analysis and Visualization Platform. Expressions were presented as log2-transformed data. Statistical analyses were performed by the R2 Platform using one-way ANOVA. ** *p* < 0.01; *** *p* < 0.001.

TMPRSS2-ERG fusion plays important roles in PC initiation and progression [7,8]. This might be particularly relevant as the fusion is induced in LNCaP cells by androgen signaling [53] and the derivation of the LE genes from LNCaP cells (Figure 2A). The observed differential expressions of TMEM45B, C1orf106, and SRD5A3 in iCluster 2 PCs, which are enriched with TMPRSS2-ERG fusion (Figure 3A), indicate a relationship of these LE genes with ERG fusion. By using the Sueltman dataset [54] within the R2: Genomics Analysis and Visualization Platform (http://r2.amc.nl http://r2platform.com accessed on 19 December 2020) website, significant increases (*p* < 0.05) of TMEM45B, ARFGEF2, GPR110, and PTPN3 in PCs with TMPRSS2-ERG fusion were demonstrated (Figure 4). Trends (*p* < 0.1) of differential expression in PCs with TMPRSS2-ERG fusion compared to those without the fusion also occur in NANS, C1orf106, FBOX6, and SRD5A3 (Figure 4).

By using the Sawyers dataset [55] within the R2: Genomics Analysis and Visualization Platform (http://r2.amc.nl http://r2platform.com accessed on 19 December 2020) website, we demonstrated upregulations of TMEM45B, FBOX6, and SRD5A3 in mPCs compared to primary PCs along with downregulations of NANS, LY75, and ARFGEF2 in mPCs in comparison to primary PCs (Figure 3B).

**Figure 5:**
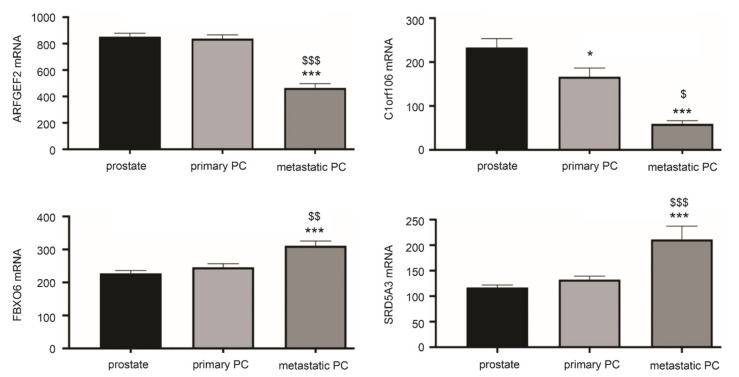
Differential expression of LE genes following PC progression. The curated GEO GDS2546 dataset was used for these analyses. Normal prostate tissue (prostate) *n* = 67, primary PCs (*n* = 66), and mPCs (*n* = 25) [56,57]. * *p* < 0.05, *** *p* < 0.001 in comparison to normal prostate tissues; $ *p* < 0.05, $$ *p* < 0.01, $$$ *p* < 0.001 in comparison to primary PCs. One-way ANOVA was performed, followed by post-hoc analysis using the Tukey’s multiple comparisons test.

The above analyses reveal potential differential expression of LE genes in PC initiation using the TCGA dataset within the GEPIA2 database and PC progression from primary tumors to distant metastasis using the Sawyers dataset with the R2 Genomics. A limitation of these analyses was the use of different datasets for PC initiation and progression analysis respectively. To further determine the differential expressions, we examined LE gene expression using a curated GEO dataset: GDS2546 (*n* = 167) which contains normal prostate tissues (*n* = 76), primary PCs (*n* = 66), and mPCs (*n* = 25) [56,57]. By using this dataset, downregulations of C1orf106 in PC compared to normal prostate tissues and mPCs compared to primary PCs were observed (Figure 5). ARFGEF2 was downregulated in mPCs compared to primary PCs; both FBOX6 and SRD5A3 are upregulated in mPCs (Figure 5). These alterations are consistent with those observed using the TCGA and Sawyers datasets (Figure 3). Taken together, these analyses convincingly demonstrate differential expression of LE genes following PC development.

### 3.3. Differentional Expression of CNTN1 and LE Genes in CRPC

**Figure 6:**
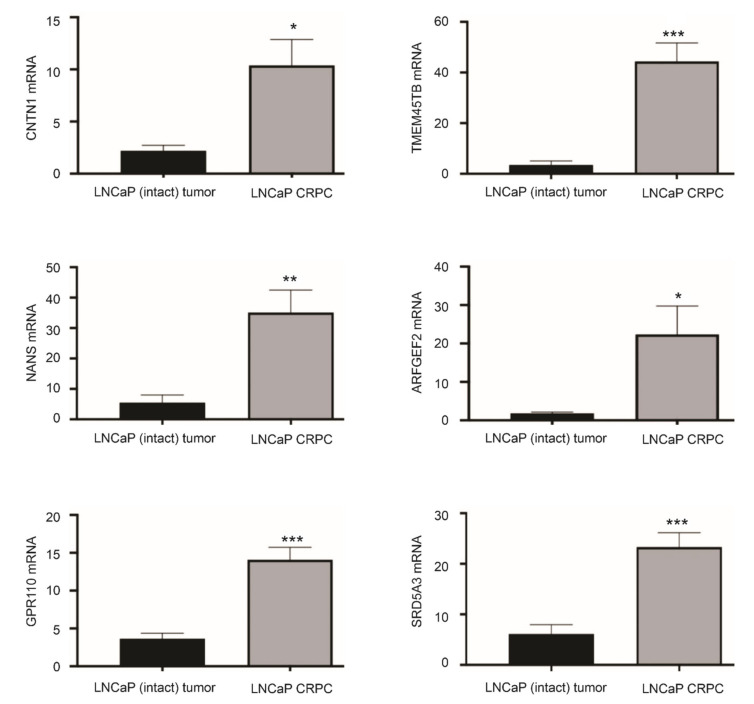
Differential expression of LE genes in LNCaP CRPCs compared to androgen-sensitive LNCaP tumors. LNCaP cells were used to produce xenografts in intact NOD/SCID or castrated animals (CRPC). The expression of the indicated genes was determined by real-time PCR in LNCaP intact tumors (*n* = 6) and LNCaP CRPCs (*n* = 5). * *p* < 0.05; ** *p* < 0.01, *** *p* < 0.001 in comparison to LNCaP EV tumors. Statistical analyses were performed using Student t-test (2-tails).

The enrichment of the CREIGHTON_ENDOCRINE_THERAPY_RESISTANCE_3 gene set (Figure 2A) with a role in resistance of breast cancer to endocrine therapy [46] indicates differential expression of CNTN1 and the LE genes in CRPCs. To examine this possibility, we have generated LNCaP xenografts in intact (*n* = 6) and castrated (*n* = 5) NOD/SCID mice. The development of LNCaP tumors resistant to castration (CRPC) was confirmed by tumor regrowth and resurge of serum PSA [41]. In comparison to LNCaP tumors produced in intact mice, LNCaP CRPCs display upregulations of CNTN1 along with five LE genes: TMEM45B, NANS, ARFGEF2, GPR110, and SRD5A3 (Figure 6). These observations are novel and support the concept of potential differential expression of LE component genes following PC progression.

### 3.4. The LE Genes Predicting PC Relapse

**Figure 7:**
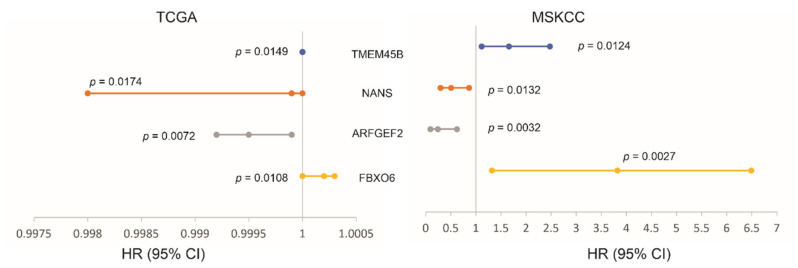
Associations of the indicated LE genes with prostate cancer relapse in The Cancer Genome Atlas (TCGA) PanCancer (*n* = 492) and MSKCC (*n* = 140) cohorts. Univariate Cox PH (proportional hazard) analyses were performed using R survival package. The PH assumption was violated for FBXO6 in the MSKCC cohort; the assumption for others was confirmed. The FBXO6 expression in the MSKCC cohort was presented as log2 transformed data. HR and 95% CI for TEME45B in the TCGA dataset are 1, respectively.

**Figure 8:**
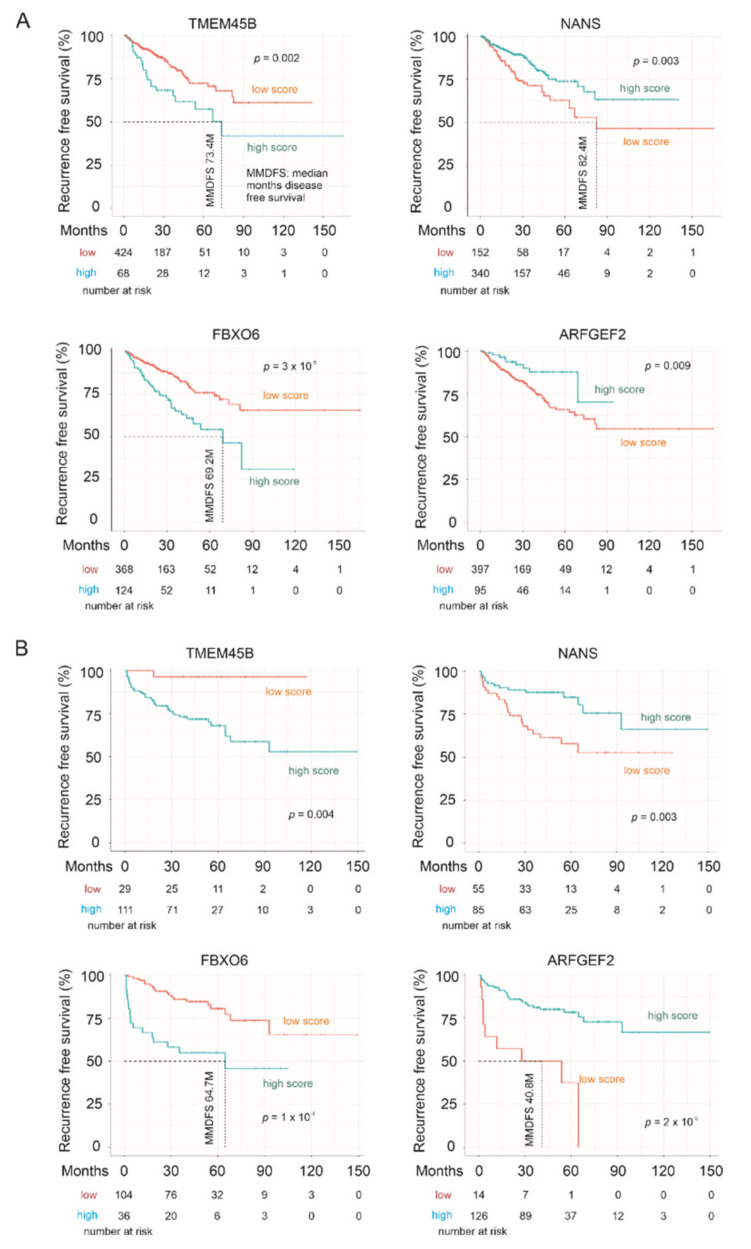
LE gene expressions stratify the risk of prostate cancer recurrence. The Kaplan–Meier survival curves for the indicated genes were produced with the R survival package using data from the TCGA PanCancer (**A**) and MSKCC (**B**) cohorts. Cutoff points for individual genes were obtained using the R Maxstat package. Statistical analyses were performed using logrank test.

**Figure 9:**
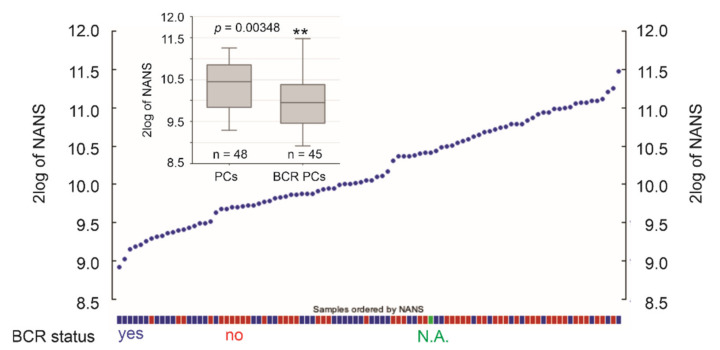
Downregulation of *NANS* expression in primary PCs with BCR development. *NANS* mRNA expression in individual PCs within the Sueltman dataset are shown; the status of PC with and without BCR development is indicated. The illustrations were produced using the R2: Genomics platform. ** *p* < 0.01.

We subsequently analyzed the associations of LE genes with PC relapse or biochemical recurrence (BCR) following curative therapies. The TCGA PanCancer and MSKCC datasets within cBioPortal were extracted and analyzed. Among the LE genes, upregulations of TMEM45B and FBXO6 as well as downregulations of NANS and ARFGEF3 are associated with PC relapse in the TCGA cohort (*n* = 492) and MSKCC cohort (*n* = 140) (Figure 7). In both independent cohorts, all four genes significantly stratify PCs with high relapse risk from those with low relapse risk (Figure 8). While tumors within the group marked with high expression of either TMEM45B or FBXO6 are at higher risks of relapse, the reverse patterns are observed for NANS and ARFGEF3 (Figure 8). The correlations of low NANS expression with PC recurrence in both TCGA and MSKCC cohort (Figure 8) are in accordance with the reduced NANS expression in primary PC with BCR occurrence compared to those without BCR in the Sueltman dataset [54] (Figure 9). Collectively, data from multiple independent cohorts supports the biomarker values of TMEM45B, FBXO6, NANS, and ARFGEF3 in predicting PC recurrence.

### 3.5. Effective Prediction of PC Recurrence Using LE Genes as a Multigene Panel

**Figure 10:**
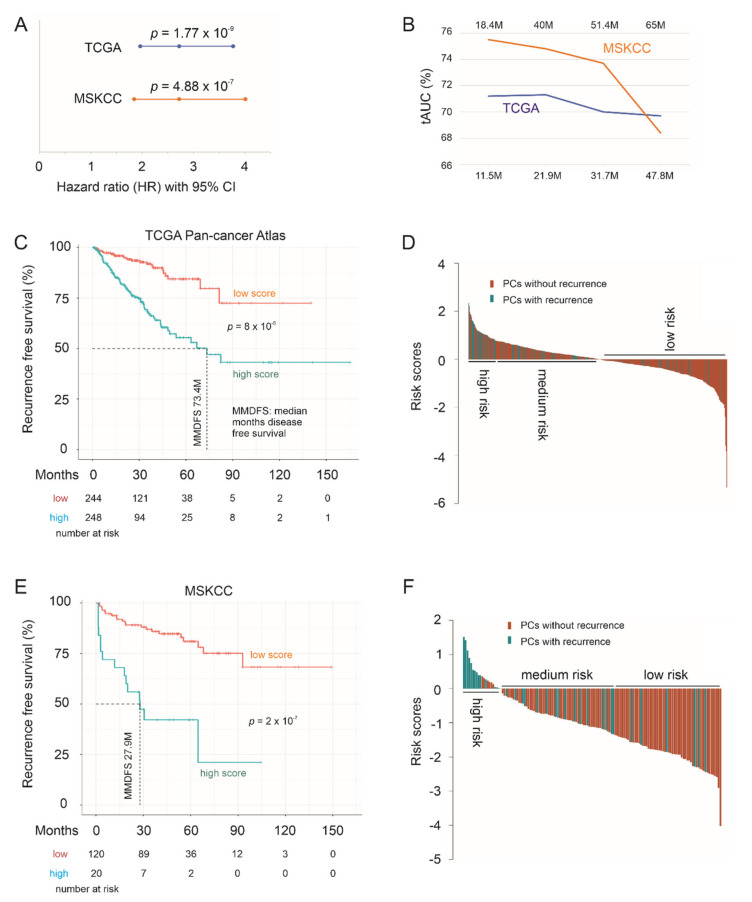
The LE gene panel robustly predicts PC recurrence. (**A**) hazard ratio (HR), 95% CI, and *p* values for LE panel scores in predicting PC relapse. (**B**) Time-dependent receiver operating characteristic (ROC) curve. The time scales above and below are for the MSKCC and TCGA PanCancer cohort, respectively. (**C**–**F**) Kaplan–Meier survival curves and the risk score waterfall plot are for the TCGA (**C**,**D**) and MSKCC (**E,F**) cohort. For waterfall plots, the cutoff points were used as the baselines. Statistical analyses were performed using log rank test.

We subsequently determined the biomarker potential of the LE genes in assessing PC relapse risk as a multigene panel. The panel scores for individual PCs were produced using the formula: ∑(f_i_)_n_ [f_i_: Cox coefficient (coef) of gene_i_ × gene_i_ expression, *n* = 10], where Cox coefs for individual genes were obtained using the multivariate Cox model. The panel scores robustly predict PC relapse at HR 2.72, 95% CI 1.96–3.77, *p* = 1.77 × 10^−9^ in the TCGA PanCancer cohort and HR 2.72, 95% CI 1.84–4.01, *p* = 4.88 × 10^−7^ in the MSKCC cohort (Figure 10A). The scores discriminate PCs with recurrence and those without a time-dependent receiver operating characteristic (tROC) AUC values of 71.2% at 11.5 months in the TCGA cohort and 75.5% at 18.4 months in MSKCC cohort (Figure 10B). With cutoff points optimized using R Maxstat package, the LE gene panel scores effectively separate PCs into a high- and low-relapse risk group in both the TCGA PanCancer and MSKCC cohort (Figure 10C,E). Additionally, the LE genes as a panel is substantially more powerful in predicting PC relapse compared to its individual component genes (comparing Figure 7 and Figure 8 with Figure 10). Using risk score waterfall plot, PCs in both cohorts can be grouped into a high-risk, medium-risk, and low-risk group with respect to recurrence (Figure 10D,F). Collectively, these analyses reveal an attractive potential of the LE genes as a multigene panel in assessing PC relapse risk.

### 3.6. The LE Panel as an Independent Risk Factor of PC Recurrence

PC relapse risk is associated with clinical features such as tumor stage, Gleason score (GS)-based WHO PC grading system, and margin status [36,58]. As expected, these clinical features are risk factor of PC relapse (Table 2, see univariate Cox analysis). The LE gene panel score remains a predictor of PC recurrence after adjusting for age at diagnosis, tumor state, WHO PC grades, and margin status (Table 2). Among the 10 LE genes, ARFGEF2 remains predictive to PC recurrence after adjusting for these clinical factors at HR 0.9996, 95% CI 0.99925–0.9999, and *p* = 0.0219; this is interesting considering its single gene status.

### 3.7. LE Gene Panel Predicts Poor Overall Survival (OS) in Renal Cell Carcinoma (RCC) and Bladder Carcinoma

**Figure 11:**
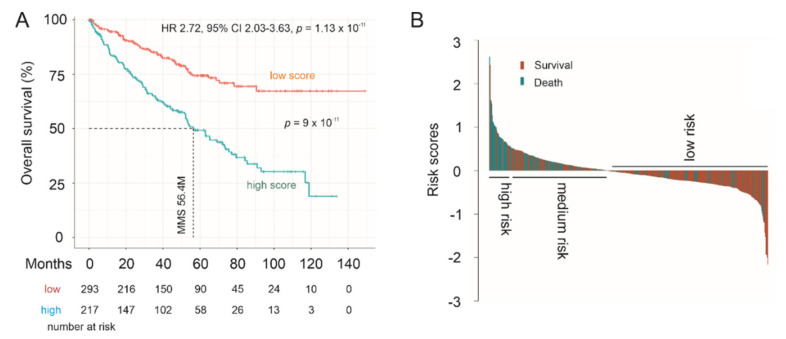
Prediction of poor Overall Survival (OS) of clear cell renal cell carcinoma (ccRCC) using the LE multigene panel. (**A**) Kaplan–Meier survival curves. Statistical analyses were performed using log rank test. (**B**) Waterfall plot of risk score.

PC is a urogenital malignancy, under which also includes kidney and bladder cancer. It is of interest to note that the LE panel scores robustly predict poor OS in clear cell RCC (ccRCC) using the TCGA PanCancer ccRCC dataset at HR 2.72, 95% CI 2.03–3.63, *p* = 1.12 × 10^−11^ (Figure 11A). The LE panel scores were redefined using the TCGA PanCancer ccRCC dataset with the multivariate Cox model. Nonetheless, the observed predictive effectiveness towards ccRCC poor OS is comparable to the Panel’s prediction of PC relapse at HR 2.72, 95% CI 1.96–3.77, *p* = 1.77 × 10^−9^ in the TCGA PanCancer PC cohort (Figure 10A). In line with this concept, the Panel scores effectively stratifies ccRCC fatality risk (Figure 11A) and the Panel risk scores clearly separate ccRCCs into a high-, medium-, and low-risk group based on the fatality risk (Figure 11B). The stratification efficiency was comparable to LE gene panel score in stratification of PC recurrence risk in both the TCGA PanCancer PC and MSKCC cohorts (comparing Figure 11 to Figure 10C,D and Figure 10E,F). Furthermore, LE gene panel score remains a risk factor of ccRCC death after adjusting for age at diagnosis, sex, tumor stage, and tumor grade (Table 3). The TCGA PanCancer ccRCC dataset has been well-demonstrated for its suitability in OS biomarker studies [44]. As the LE multigene panel was derived based on its association with CNTN1 in PC, the observed biomarker potential of the LE gene panel in assessing both poor OS in ccRCC and in predicting PC relapse risk is intriguing. This supports the potential role of the LE multigene panel in assessing PC recurrence.

**Figure 12:**
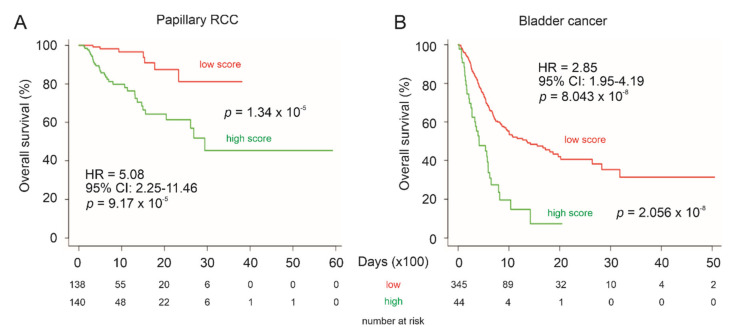
Prediction of poor OS of pRCC and bladder cancer using the LE multigene panel. Kaplan–Meier survival curves of pRCC (**A**) and bladder cancer (**B**). The TCGA pRCC (*n* = 278) and bladder cancer (*n* = 390) datasets were organized by SurvExpress. Risk scores of LE panel were calculated using multivariate Cox fitting; cutoff points were optimized to stratify low-risk and high-risk populations.

To further support LE gene panel’s prognostic biomarker values in urogenital cancers, we analyzed the panel’s potential in the stratification of low and high mortality risk groups of papillary RCC (pRCC), the second common type of RCC [59], and bladder cancer. For this analysis, the SurvExpress website (http://bioinformatica.mty.itesm.mx:8080/Biomatec/SurvivaX.jsp accessed on 19 December 2020) was used. The program calculates a multigene panel risk score using the multivariate Cox system [60] which is the system used here. The LE panel significantly separates low-risk and high-risk pRCC (Figure 12A) and bladder cancer groups (Figure 12B) as well as predicts fatality risk, evident by the HR values (Figure 12).

### 3.8. LE Gene Panel Predicts Poor OS in CNTN1-Associated Cancer Types

**Figure 13:**
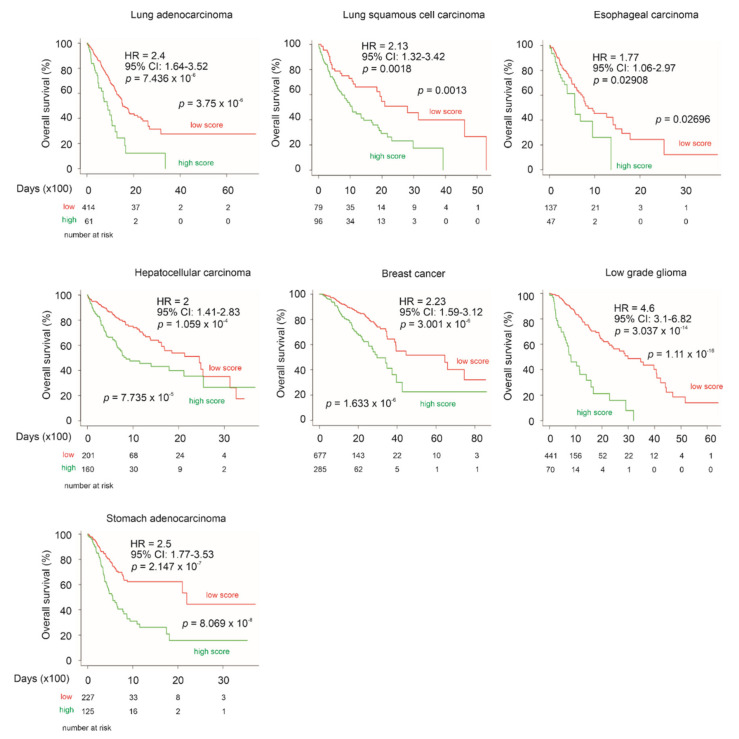
LE gene panel predicts poor OS in multiple cancer types with reported relationship with CNTN1 expression. The above datasets were organized by SurvExpress; specifically, lung adenocarcinoma (*n* = 475), lung squamous cell carcinoma (*n* = 175), esophageal carcinoma (*n* = 184), hepatocellular carcinoma (*n* = 361), breast cancer (*n* = 962), low grade glioma (*n* = 512), and stomach adenocarcinoma (*n* = 352) were all from TCGA. HR and 95% CI (confidence interval) for individual analyses are included. Kaplan–Meier survival curves are shown. Statistical analyses were performed using log rank test. All analyses were carried out using tools provided by SurvExpress.

CNTN1 has been reported to associate with poor prognosis in patients with lung, esophageal and oral squamous cell carcinomas [26,27,28], and hepatocellular carcinoma [29]. CNTN1 upregulation was reported to correlate with worse clinical features in breast cancer [30], astrocytic glioma [31], thyroid cancer [32], and stomach cancer [33]. To further analyze the prognostic biomarker values of the LE gene panel, we have determined the panel’s ability to stratify the fatality risk of these cancer types using datasets organized by SurvExpress. Although the fatality event occurred in a small number (*n* = 14) in a thyroid cancer cohort (*n* = 489), LE gene panel risk score was able to significantly stratify low-risk and high-risk groups (logrank test *p* = 4.8 × 10^−4^). The panel risk scores predict poor OS in lung adenocarcinoma, lung squamous cell carcinoma, esophageal carcinoma, hepatocellular carcinoma, breast cancer, low grade glioma, and stomach adenocarcinoma, evident by the respective HR values (Figure 13). The LE gene panel risk scores can significantly separate patients with the individual cancer types into the respective low-risk and high-risk populations (Figure 13). The prediction of poor OS is particularly effective in low grade glioma and stomach adenocarcinoma (Figure 13). Collectively, these analyses strengthen the biomarker potential of the LE gene panel.

## 4. Discussion

Mechanisms underlying PC initiation, progression, and therapy resistance have been extensively investigated. Risk assessment for PC prognosis using clinical features and molecular biomarkers has been actively pursued, leading to numerous biomarker candidates particularly for BCR risk assessment [61]. However, even with such extensive investigations, our understanding of PC progression remains incomplete and current prediction of PC relapse risk still needs substantial improvement. Effective prognostic biomarkers are the basis for therapy decision making and individualized therapies.

Our research contributes to this effort. Our initial attempt in the characterization of DEGs relative to CNTN1 overexpression in LNCaP cells led to the identification of an enriched gene set: CREIGHTON_ENDOCRINE_THERAPY_RESISTANCE_3. The enrichment analysis was performed using a recently-developed FGSEA platform (https://www.biorxiv.org/content/10.1101/060012v2.full accessed on 19 December 2020), a system similar to the most popular GSEA system developed by the Broad Institute [45]. However, gene set enrichment analysis is understandably associated with numerous limitations, including the lack of gold standard dataset to capture the complex nature of gene expression, a single-gene analysis method (the most popular one), and others [62,63]. This study has additional limitations. The functionality of CNTN1 in CRPC development in vitro and in vivo needs to be investigated; some LE genes display differential expression in PC vs. normal prostate tissues (Figure 3A), mPCs vs. local PCs (Figure 3B), TMPRSS2-ERG+ vs.TMPRSS2-ERG+ (Figure 4), as well as following a course of disease progression (Figure 5). While these LE genes have a relationship with CNTN1 (Figure 2A), CNTN1 mRNA expression is complex. The curated GEO GDS2546 dataset used to examine LE gene expression following a PC course (Figure 5) did not support a similar analysis on CNTN1 mRNA. While CNTN1 mRNA expression is significantly increased in TMPRSS2-ERG+ PCs compared to the negative counterparts, its mRNA expression is reduced in PCs vs. prostate tissues and mPCs vs. local PCs. These analyses may present a mixed message regarding CNTN1′s association with PC progression. Nonetheless, mRNA expression status is not equivalent to gene’s physiological roles mediated by proteins. For instance, whilst CNTN1 mRNA expression does not correlate with PC recurrence, its protein expression was reported to associate with PC relapse [34]. Importantly, CNTN1 promotes PC metastasis [34]. CNTN1-facilitated PC progression was also observed by others [35]. Despite a complex relationship between CNTN1 and the LE genes, our analyses nonetheless clearly reveal the novel and robust prognostic biomarker potentials of LE genes not only in PC but also in other cancer types.

The leading proportion of the enriched CREIGHTON_ENDOCRINE_THERAPY_RESISTANCE_3 gene set robustly predicts PC relapse risk in two independent datasets, the TCGA PanCancer PC (*n* = 492) and MSKCC (*n* = 140) cohorts. The prognostic biomarker properties of this 10-gene panel are further validated by its comparable competency in predicting ccRCC poor OS. Although this leading-edge (LE) panel was derived from PC research, its prognostic biomarker potential towards ccRCC is certainly interesting and is likely attributable to the commonality shared by PC and ccRCC with both being urogenital cancers. In support of this concept, the LE gene panel significantly predicts fatality risk in other urogenital cancers, papillary RCC and bladder cancer (Figure 12). Similar observations were reported in DNA methylation biomarkers of urogenital cancers [64]. Furthermore, the LE gene panel displays prognostic prediction in other cancer types with which CNTN1 expression has been reported to associate with either poor prognosis or cancer severity (see Section 3.8 for details), including lung cancer, esophageal carcinoma, hepatocellular carcinoma, breast cancer, low grade glioma, and stomach adenocarcinoma (Figure 13). Collectively, we provide a comprehensive set of evidence supporting the LE gene panel as a novel prognostic biomarker for multiple cancer types. The LE gene panel as a multigene panel prognostic biomarker is novel to PC.

The novelty of this multigene panel is also attributed to its component genes. Among the 10 individual genes, 5 genes have not been reported or with articles published in PC, including C1orf106 and FBXO6 (Table 1). Downregulations of C1orf106 occur in PCs compared to prostate tissues, PCs with TMPRSS2-ERG fusion compared to those without the fusion, and mPCs compared to primary PCs (Figure 3A, Figure 4 and Figure 5). C1orf106 plays a role in activating innate immunity in breast cancer [65], suggesting that its downregulation contributes to the evasion of immune checkpoints during PC initiation and progression. In view of the emerging roles of escaping immune surveillance in all stages of tumorigenesis, the potential contributions of C1orf106 to this aspect of PC certainly warrants further investigation in future. Reductions in ARFGEF2 expression in mPCs compared to primary tumors were observed in two independent cohorts (Figure 3B and Figure 5). In both TCGA PanCancer and MSKCC cohorts, ARFGEF2 downregulations increased the risk of PC relapse (Figure 7) and this biomarker value is independent of age at diagnosis, tumor stage, WHO PC grade, and surgical margin status. The oncogenic actions of ARFGEF2 remain unclear not only in PC but also tumorigenesis in general with *n* = 4 articles in PubMed under “ARFGEF2 and Cancer”. Our research here indicates that ARFGEF2 downregulation may facilitate PC progression. FBXO6 was upregulated in mPCs over primary PCs in two independent datasets (Figure 3B and Figure 5). FBXO6 can induce Chk1 degradation [66]. Chk1 plays an essential role in maintaining genome stability via contributing to activation of cellular DNA damage response [67]. FBXO6 might contribute to genome instability during PC progression; genome instability is an essential oncogenic factor of PC and other cancer types [68].

Among four genes (*TMEM45B*, *NANS*, *GPR110*, and *SRD5A3*) with known associations with PC (Table 1), their contributions to PC, except SRD5A3, remain largely unclear. PubMed listed *n* = 1 and *n* = 2 articles under “TMEM45B and Prostate Cancer” and “NANS and Prostate Cancer” respectively. There were also two publications on the topic of “GPR110 and Prostate Cancer” in PubMed. We provide a comprehensive set of evidence revealing upregulation of TMEM45B and NANS in PC initiation (Figure 3A), progression to mPCs (Figure 3B), their association with TMPRSS2-ERG fusion (Figure 4), progression to CRPC (Figure 6), and their biomarker value in predicting PC recurrence in two-independent datasets (Figure 7). Collectively, our research significantly strengthens the involvement of both TMEM45B and NANS in PC development. Their contributions to PC initiation and progression should be investigated in future.

For the rest of LE genes, their contributions are critical for the LE gene panel’s effectiveness in predicting PC release, although their differential expression following PC pathogenesis and their biomarker values as individual genes were not significant. This enhancement properties are likely attributable for these genes being components of the CREIGHTON_ENDOCRINE_THERAPY_RESISTANCE_3 gene set.

While this study advances the current understanding of CNTN1 in PC, we like to emphasize the complex nature of these LE genes with respect to their contributions to CNTN1-mediated PC pathogenesis. This interpretation is based on the upregulation of these LE genes in LNCaP CNTN1 cells over the LNCaP EV cells and their differential expressions are not always positively correlated with PC progression. For instance, C1orf106 is consistently downregulated following PC initiation and metastatic progression. However, it is not uncommon for tumor suppressor genes to be upregulated in response to its tumor surveillance role following oncogenesis [69,70].

## 5. Conclusions

We provide the first demonstration of CNTN1 upregulation in CRPC in vivo (Figure 6), which extends the reported CNTN1 oncogenic functions in PC [34,35]. This observation was further supported by the enrichment of a gene set facilitating breast cancer resistance to endocrine therapy (Figure 2A). The 10 leading-edge genes can predict PC recurrence with a high level of certainty in two independent cohorts (Figure 10). These LE genes consist of genes that are either under-examined or novel in PC (Table 1). For the former four genes (*TMEM45B*, *NANS*, *GPR110*, and *SRD5A3*), we demonstrated upregulations of TMEM45B, NANS, and SRD5A3 in PC initiation and metastatic progression as well as significant increases of all four genes in LNCaP cell-derived CRPCs (Figure 3 and Figure 6). For the five genes novel to (unknown or with function not clear to PC, see Table 1), C1orf106 exhibits consistent differential expression in PCs vs. normal prostate tissues, PCs with or without TMPRSS2-ERG fusion, and mPCs compared to primary PC (Figure 3, Figure 4 and Figure 5). Consistent with ARFGEF2 protein expression being detected in a subclass of bone metastatic CRPCs [52], we observed its differential expression in PCs with TMPRSS2-ERG fusion vs. those without the fusion (Figure 4), mPCs vs. primary PCs (Figure 3B), as well as CRPC vs. androgen-sensitive PCs (Figure 6). Collectively, this investigation reveals multiple novel observations that might be relevant to biological processes underlying PC initiation and progression.

## Figures and Tables

**Table 2:** Univariate and multivariate Cox analysis of LE gene panel for PC relapse.

Factors	Univariate Cox Analysis			Multivariate Cox Analysis		
	HR	95% CI	*p*-Value	HR	95% CI	*p*-Value
LE Panel ^1^	2.72	1.96–3.37	1.77 × 10^−9^ ***	1.91	1.37–2.671	0.000132 ***
Age ^2^	1.02	0.99–1.05	0.189	0.99	0.96–1.02	0.6201
WHO IV ^3^	9.76	1.28–74.6	0.0282 *	6.32	0.81–49.55	0.079158
WHO V ^3^	21.38	2.96–154.5	0.00241 **	10.28	1.36–77.77	0.024047 *
Tstage 1 ^4^	3.69	2.08–6.52	7.45 × 10^−6^ ***	1.61	0.84–3.09	0.151878
Margin 1 ^5^	2.30	1.52–3.48	8.1 × 10^−5^ ***	1.32	0.84–2.08	0.228092

^1^ LE gene panel score; ^2^ Age at diagnosis; ^3^ Compared to WHO PC grade I, WHO II and WHO III are not significant at univariate Cox analysis; ^4^ Tstage (T stage-based tumor stage) 1: tumor stage 1 (3 + 4) in comparison to Tstage 0 (tumor stage 1 + 2); ^5^ Surgical margin 1 compared to surgical margin 0; HR: hazard ratio; CI: confidence interval. *, **, *** for *p* < 0.05, 0.01, and 0.001, respectively.

**Table 3:** Univariate and multivariate Cox analysis of LE gene panel for poor OS (overall survival) of ccRCC (clear cell renal cell carcinoma).

Factors	Univariate Cox Analysis			Multivariate Cox Analysis		
	HR	95% CI	*p*-Value	HR	95% CI	*p*-Value
LE Panel ^1^	2.72	2.03–3.63	1.13 × 10^−11^ ***	1.67	1.18–2.35	0.00341 **
Age ^2^	1.03	1.02–1.04	2.78 × 10^−6^ ***	1.03	1.02–1.05	3.68 × 10^−5^ ***
Sex ^3^	0.96	0.70–1.31	0.793	0.96	0.696–1.329	0.81224
Stage III ^4^	2.80	1.84–4.23	1.28 × 10^−6^ ***	2.05	1.32–3.16	0.00127 **
Stage IV ^4^	6.83	4.60–10.12	<2 × 10^−16^ ***	4.53	2.87–7.13	7.13 × 10^−11^ ***
Grade 3 ^5^	1.94	1.32–2.86	0.000753 ***	1.43	0.96–2.13	0.08017
Grade 4 ^5^	5.74	3.59–8.05	3.06 × 10^−16^ ***	2.01	1.25–3.23	0.00383 **

^1^ LE gene panel score; ^2^ Age at diagnosis; ^3^ Compared to females; ^4^ Compared to Stage I, Stage II is not significant at univariate Cox analysis; ^5^ Compared to Grade 1 + 2 (both grades were combined because of small sample number for Grade 1 samples, *n* = 12); HR: hazard ratio; CI: confidence interval. **, *** for *p* < 0.01 and 0.001, respectively.

## Data Availability

Not applicable.
